# Dendritic Cell-Based Immunotherapies to Fight HIV: How Far from a Success Story? A Systematic Review and Meta-Analysis

**DOI:** 10.3390/ijms17121985

**Published:** 2016-11-26

**Authors:** Antonio Victor Campos Coelho, Ronald Rodrigues de Moura, Anselmo Jiro Kamada, Ronaldo Celerino da Silva, Rafael Lima Guimarães, Lucas André Cavalcanti Brandão, Luiz Cláudio Arraes de Alencar, Sergio Crovella

**Affiliations:** 1 Department of Genetics, Federal University of Pernambuco, Avenida da Engenharia, Cidade Universitária, Recife 50740-600, Brazil; avccbio@gmail.com (A.V.C.C.); ronaldmoura1989@gmail.com (R.R.d.M.); anselmojiro@gmail.com (A.J.K.); rafaellg@gmail.com (R.L.G.); 2 Laboratory of Immunopathology Keizo Asami (LIKA), Federal University of Pernambuco, Avenida da Engenharia, Cidade Universitária, Recife 50740-600, Brazil; ronaldocelerino@yahoo.com.br (R.C.d.S.); lucabrand@gmail.com (L.A.C.B.); 3 Department of Pathology, Federal University of Pernambuco, Avenida Prof. Moraes Rego, 1235, Cidade Universitária, Recife 50670-901, Brazil; 4 Department of Tropical Medicine, Federal University of Pernambuco. Avenida Prof. Moraes Rego, 1235, Cidade Universitária, Recife 50670-901, Brazil; lularraes@hotmail.com; 5 Instituto de Medicina Integral Professor Fernando Figueira (IMIP), Boa Vista, Recife 50070-550, Brazil; 6 IRCCS Burlo Garofolo and University of Trieste, Via dell’ Istria 65/1, Trieste 34137, Italy

**Keywords:** human immunodeficiency virus, dendritic cell, clinical trial, vaccine, meta-regression

## Abstract

The scientific community still faces the challenge of developing strategies to cure HIV-1. One of these pursued strategies is the development of immunotherapeutic vaccines based on dendritic cells (DCs), pulsed with the virus, that aim to boost HIV-1 specific immune response. We aimed to review DCs-based therapeutic vaccines reports and critically assess evidence to gain insights for the improvement of these strategies. We performed a systematic review, followed by meta-analysis and meta-regression, of clinical trial reports. Twelve studies were selected for meta-analysis. The experimental vaccines had low efficiency, with an overall success rate around 38% (95% confidence interval = 26.7%–51.3%). Protocols differed according to antigen choice, DC culture method, and doses, although multivariate analysis did not show an influence of any of them on overall success rate. The DC-based vaccines elicited at least some immunogenicity, that was sometimes associated with plasmatic viral load transient control. The protocols included both naïve and antiretroviral therapy (ART)-experienced individuals, and used different criteria for assessing vaccine efficacy. Although the vaccines did not work as expected, they are proof of concept that immune responses can be boosted against HIV-1. Protocol standardization and use of auxiliary approaches, such as latent HIV-1 reservoir activation and patient genomics are paramount for fine-tuning future HIV-1 cure strategies.

## 1. Introduction

The adaptive immune response during viral infections is mediated by antigen-presenting cells (APC), such as macrophages, B lymphocytes and dendritic cells (DCs), which capture antigens and present them to naïve lymphocytes [1,2].

Immature DCs are characterized by high endocytic capacity, with constant sampling of the surroundings of peripheral tissues (such as mucosa and epithelia) for pathogen and injury sensing [3]. DC activation, upon pathogen sensing, leads to capture of proteins, processing into peptides and antigen presentation to lymphocytes, and they are thus considered the most potent antigen-presenting cell of the immune system [4].

**Figure 1:**
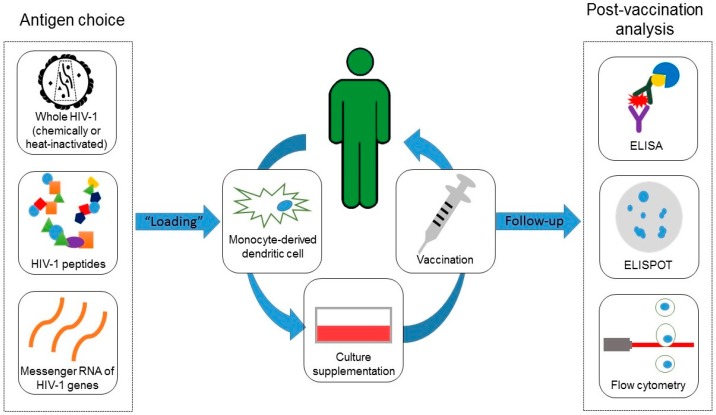
Rationale behind dendritic-cell based experimental anti-HIV-1 vaccines. Providing dendritic cells with specific antigen presentation will result in a reconditioning of host immune response against HIV-1, in the hopes that this will lead to a functional cure. The experimental vaccination protocols details vary, but they basically consist in choosing an antigen (**left** panel), such as autologous (taken from the volunteer) whole virus that are heat- or chemically-inactivated (**top left** panel), viral peptides such as gag or pol residues, for example (**middle left** panel) or even viral messenger RNA molecules (**bottom left** panel). Following antigen choice, dendritic cells must be obtained for vaccine preparation (**center** panel). Usually, leukocytes are collected through leukapheresis and monocytes are isolated and differentiated in vitro into immature dendritic cells. Then, dentritic cells (DCs) are "loaded" with the chosen antigen and activated with cytokine supplementation, for example with granulocyte-macrophage colony-stimulating factor (GM-CSF), interferon alpha and gamma (IFN-α and IFN-γ), interleukins 4 and 6 (IL-4 and IL-6) and tumor necrosis alpha (TNF-α) into mature DCs. Defined amounts of mature dendritic cells are then periodically injected in the individual. Usually, immune response is assessed before, and during vaccination follow-up, through methodologies such as enzyme-linked immunosorbent assay (ELISA), enzyme-linked immunospot (ELISPOT), flow cytometry or any combination thereof (**right** panel), to evaluate (qualitatively and quantitatively) how it changed following vaccination.

As the success of antiretroviral therapy (ART) enabled the scientific community to turn its efforts to the search for a definitive cure for HIV-1, several therapeutic DC-based strategies have been developed based on the rationale that providing DCs with specific antigen presentation would “boost” the recovery of host immune response against HIV-1 (Figure 1).

Thus, we aimed to summarize reports from clinical trials with DC-based therapeutic vaccines with a systematic review supported by meta-analysis and meta-regression modeling to gain insight from past experiences to improve future therapeutic strategies against HIV-1.

## 2. Results

### 2.1. Study Screening and Characteristics

**Figure 2:**
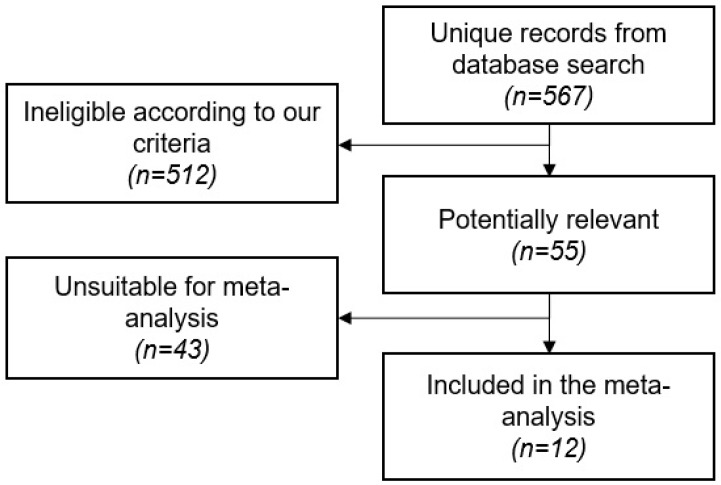
Summary of the number of screened, reviewed and included or excluded dendritic cell-based experimental anti-HIV-1 vaccines.

The search strategy resulted in a total of 567 unique abstracts, which were assessed for eligibility. A total of 55 abstracts reporting clinical trial findings were selected for further review. Among those, we extracted data from 12 studies and included them in the meta-analysis (Figure 2).

The characteristics of the 12 studies selected for inclusion in the meta-analysis are summarized in Table 1 and Table 2. The Table 1 and Table 2 deal with study design-related data from each protocol. Table 3 and Table 4 contain information concerning technical differences among the studies.

The majority of selected studies recruited ART-experienced individuals for the vaccine trials (eight out of 12 studies). Overall, the included individuals had good immunological status, as expected by the reported inclusion criteria and judged by their pre-vaccination CD4+ T cell counts (median 632 cells/mm^3^, interquartile range, IQR = 559.5–667), with only two studies reporting CD4+ T cell counts medians below 500 cells/mm^3^ [5,6]. One study did not inform the CD4+ T cell counts from their recruited patients [7].

Median number of recruited individuals was 18 (IQR = 11–25, minimum and maximum: 4 and 54, respectively). Each individual received a median of four doses from the experimental DC-based vaccine (minimum of three and maximum of six doses), with a biweekly periodicity (five out of 12 studies) or every four weeks or more (seven out of 12). The form of administration of the experimental vaccine was similar in the majority of the studies, being intradermal, subcutaneous or both, mostly on axillary areas of the body. Only one study administered the vaccine intravenously in the recruited individuals [7]. No severe vaccine-associated adverse effects were reported by any of the studies. Reported events were mostly flu-like reactions and reactions at the site of vaccine injection.

There were some differences among protocols regarding DC maturation and choice of the antigen for vaccine preparation. Five out of 12 studies loaded the DCs with (heat- or chemically-) inactivated autologous whole virus [8,9,10,11,12]. The other five loaded HIV-1 peptides [5,6,7,13,14] and only two used viral mRNA, which were electroporated into DCs for antigen production [15,16]. Cytokines used in culture medium supplementation included granulocyte-macrophage colony-stimulating factor (GM-CSF), interferon alpha and gamma (IFN-α and IFN-γ), interleukins 4 and 6 (IL-4 and IL-6) and tumor necrosis alpha (TNF-α). Other molecules for boosting DC maturation besides cytokines were: prostaglandin E2 (PGE2), lipopolysaccharide (LPS) [14], polyinosinic:polycytidylic acid [6] and CD40 ligand (CD40L) signal [16].

The median number of DCs used per vaccine dose was 7 × 10^6^ cells (minimum 0.7 × 10^6^ cells and maximum 15 × 10^6^ cells), matured in culture for a median of seven days (minimum of three days and maximum of eight days). One protocol used immature DCs, which were cultured for just two days before administration into the recruited individuals [7].

### 2.2. Meta-Analysis and Meta-Regression Results

**Figure 3:**
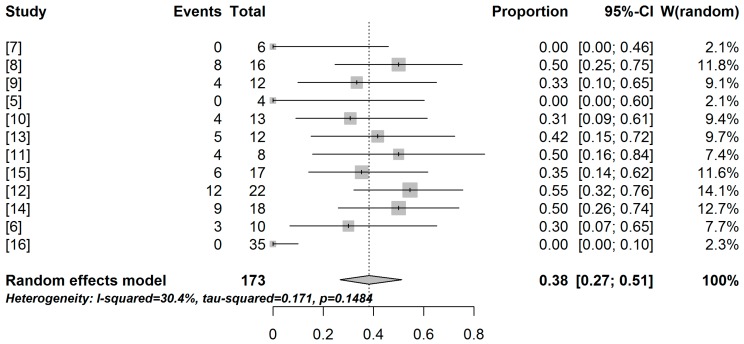
Forest plot of dendritic cell-based experimental anti-HIV-1 vaccines treatment success rate across studies. Each line represents a vaccination experimental protocol with the number of partial/transient responses to the vaccine (events column) among the vaccinated individuals (total column). The proportion of response to the vaccine is represented by a small vertical line inside a square. Each square is directly proportional to the sample size of each study, and the lines represent the 95% confidence interval. The proportions and confidence intervals are also represented in the column of the same name. The weight column (W(random)) represents how much each study contributed to the pooled sample size (*n*= 173 individuals). The pooled proportion and 95% confidence interval estimated by a random effects model meta-analysis is represented by a diamond and highlighted in bold in the lower part of the figure. Heterogeneity measures (I^2^, τ^2^) and the *p*-value of the Cochran’s Q test for heterogeneity are also represented.

The total sample number polled by the meta-analysis was 173 vaccinated individuals. The experimental vaccines had low efficiency, as suggested by the meta-analysis. The overall treatment success rate was estimated at 38.2% (95% CI = 26.7–51.3) according to a random effects model (I^2^ = 30.4%, moderate heterogeneity) (Figure 3).

The univariate pre-selection meta-regression *p*-values are summarized in Table 5. The pre-selected variables were: antigen choice (whole virus, mRNA or peptides); baseline (pre-vaccination) CD4+ T cell counts; culture medium choice (RPMI-1640, CellGro^®^, monocyte conditioned medium or X-VIVO™ 15), presence of IL-1β and IL-6 supplementation (*p* = 0.26 and *p* = 0.21, respectively) and vaccine dose periodicity (biweekly vs. every four weeks or more, *p* = 0.18).

Two variables were significantly associated with treatment success rate during univariate analysis: presence of GM-CSF supplementation was positively associated with treatment success rate (*p* = 0.003), whereas number of vaccine doses was negatively associated (*p* = 0.04) with success rate.

However, the multivariate meta-regression modeling using these variables failed to evidence any statistically significant differences that would explain the discrepancies on treatment success rate among protocols. Due to co-linearity with other variables in the model, IL-6, number of vaccine doses and vaccine periodicity were removed from the final model. This may be the consequence of the small number of observations (just 12 studies) relative to the number of considered variables (14 total, nine pre-selected). The removal has been necessary to avoid introducing bias in the model predictions. The final best-fit model is summarized in Table 6. No variables influenced the overall treatment success rate with statistical significance.

## 3. Discussion

### 3.1. Immune Responses Elicited by the Experimental Vaccines

The authors usually assessed the immune response in each experimental vaccine study by comparing pre-vaccination and post-vaccination peripheral blood mononuclear cells (PBMC) samples to assess cytokine expression and cytotoxic T lymphocytes (CTL) activity by standard techniques such as enzyme-linked immunosorbent assay (ELISA) [7,16], intracellular cytokine staining followed by flow cytometry analysis [6,8,13,14,16], Luminex^®^ multiplex bead-based cytokine assay [14] or IFN-γ enzyme-linked immunospot (ELISPOT) assays [5,10,11,12,14,15]. Some authors additionally assessed HIV-1–specific CD4+ lymphoproliferative responses [7,9,10,11,12,15].

Vaccinated individuals’ immune responses were not uniform across trials. Most, if not all studies reported that the vaccine elicited CTL responses, but only in some individuals [5,7,10,13]. Some studies showed that monocyte-derived DC from ART patients produced lower levels of IL-12 (a potent Th-1 response cytokine) after CD40L induction [17], while IL-12 reduced levels are also associated with no viral load control after DC-vaccination [6]. DCs derived from patients under ART induce higher IL-12 production under the IFN-γ and CD40L combination [17] while CD8+ cells produced more IFN-γ after DC treatment with TLR-3 ligand poly(I:C) and CD40L [18], which highlights the importance of pre-vaccination conditions in regulation of DC function.

Some authors acknowledge that these CTL and lymphoproliferative responses were in general weak and/or transient [9,11,13] or even not significant [10], but were associated with partial viral load control [8,9,11,12,13]. Other authors observed significant post-vaccination ELISPOT responses or a significant increase of anti-HIV-1 specific CD8+ T-cell activity as assessed by flow cytometry, these variants being associated with lower plasmatic viral loads levels during ATI [10,14]. Otherwise, other studies showed that memory CTLs with boosted effector activity were not associated with viral load control [6]. Regarding humoral immune response, some authors did not observe any significant post-vaccination change in total anti-HIV-1 antibody titers [8], nor in serum neutralizing activity [11].

This response heterogeneity possibly reflects the variability among the experimental vaccine protocols and individuals’ characteristics. However, the authors in general agree that the DC-based experimental vaccines had at least some immunogenicity [12,15], as showcased by the Lu et al. [8] protocol, which promoted a prolonged partial viral load control (reduction of 90% of median plasmatic viral load over the first year post-vaccination) in eight subjects among 18 chronically-infected ART-naïve recruited Brazilian individuals. This was a non-controlled, non-randomized study, but had nevertheless promising results. We chose to not include immunogenicity as an outcome in the meta-regression analysis because we were not sure how this would be performed, since the studies used several methodologies for immunogenicity assessment; thus, they cannot be compared directly. Therefore, we must regard immunogenicity only on descriptive terms, which is unfortunate, but we would introduce bias if we attempted to include them as outcomes in the meta-regression.

Overall treatment success rate was suboptimal, and the observations showed that the vaccines provide insufficient immune boosting. Some authors noted that the vaccines need to generate a specific immune response that is effective against HIV and at the same time do not generate “counter-effective” immune activation that favor virus replication instead [13].

### 3.2. The Role of Host Genomic and Trascriptomic Background

An important aspect that was not taken in consideration in all protocols was the host genetic background. Among the 12 studies included in the meta-analysis, only five reported human leukocyte antigens (HLA) alleles from the vaccinated individuals. Two studies recruited only HLA-A*02:01-positive individuals [7,13] and they were the majority in the Lu et al. study [8] (10 patients among the 18 recruited were HLA-A*02:01-positive). The four Japanese patients in the Ide et al. study [5] were A*24:02-positive and Levy et al. [14] did not specify their recruited individuals’ exact genotypes, only reporting that they were B27, B57 negative. Overall, no studies reported detailed HLA-B genotypes.

Since the majority of HLA-A*02:01 carriers exert selective pressure in epitope diversity due to the higher affinity of CTLs to dominant epitope, some authors suggested that focusing research on subdominant HIV-1 epitopes may help the development of simultaneous targeting of multiple of epitopes [19]. Therefore, HLA-typing in prospective volunteers may even help “personalization“ of HIV-1 therapeutic vaccines for each HLA make-up across worldwide populations (some collaborative initiatives may help in this endeavor, such as HLA allele frequency databases [20]), or guide peptide optimization to improve antigenicity [13,21], since some HLA alleles confer protection against HIV disease progression [22], and thus possibly influence CTL-specific/vaccine response.

Variations in genes involved in immune modulation could be an additional reason for the efficacy variability of the reviewed experimental procedures. The first study to screen the host genetic background under DC immunotherapy analyzed 768 tag and coding single nucleotide polymorphisms (SNPs) of 146 innate immunity genes and showed an association between *MBL2* rs10824792 with a weak response and *NOS1* rs693534 SNPs with a durable response to DC immunotherapy [23]; the second study considered 22 polymorphisms in 13 HIV-1 host restriction factor genes (*APOBEC3G*, *CCL4*, *CCL5*, *CCR5*, *CUL5*, *CXCR6*, *HLA-C*, *IFNG*, *PARD3B*, *Prox1*, *SDF-1*, *TRIM5*, *ZNRD1*), finding the *PARD3B* rs11884476 SNP was associated with good response DC immunotherapy [24]. Finally, a more recent genome-wide analysis evidenced that a SNP in *CNOT1* gene (rs7188697, A→G), which codes for a protein involved in the regulation of inflammatory responses and is part of a protein complex that interferes with HIV-1 replication, was associated with poor response to the experimental immunotherapy. Although these findings were limited to the 18 analyzed subjects, host genomics should be taken in consideration during HIV-1 experimental vaccine protocols, DC-based or not, to help understand its efficacy and possibly discover new therapeutic approaches [25].

A recent study indicated that the host genome contributes to the success of DC immunotherapy from two different clinical trials performed in Brazil [8] and Spain [12]. The restriction factor *TRIM22* rs7935564 G allele was associated with better response to DC-based immunotherapy in patients in both clinical trials and was also more frequent in long term non-progressors (LTNPs), thus leading to hypothesize a role for the G allele in the control of virus replication [26]. This intriguing result, although preliminary, will prompt future analyses on the contribution of host genomics in the modulation of the multifactorial response to DC-based immune therapy.

Besides genotyping, other molecular biology techniques are being employed in HIV-1 DC-based therapy research. A recent analysis evaluated gene expression profiles from PBMCs obtained from individuals vaccinated in the Allard et al. phase I/II clinical trial [15] and compared their transcriptome profile with HIV-negative controls, melanoma-affected individuals receiving melanoma DC-based immunotherapy and individuals who received seasonal influenza vaccination, showing that the vaccinated individuals transcriptome shifted to a higher expression of genes involved in cellular stress and innate immune response, which was sustained for at least 40 weeks after ATI [27]. This result highlights the importance of genomics-era analysis for the discovery of how individuals’ organisms respond to vaccines, which could lead to insights for the improvement of current strategies or the development of new ones.

### 3.3. Recommendations for Future Protocols

A difficulty encountered during our analysis was that several aspects of the protocols were not standardized, principally vaccine response criteria. Therefore, future clinical trials would benefit from standardization. The DC-based immune therapy community is actively debating protocols and elaborated a tentative consensus published elsewhere [28], which accounts for vaccine preparation methods, virus inactivation, patient follow-up and many more inherent protocol variables.

For example, the included protocols with both naïve and ART-experienced individuals, and in the case of ART-experienced individuals, each protocol evaluated vaccine efficacy differently during ATI. The non-uniformity of efficacy criteria during ATI in HIV-1 experimental trials is an issue recently reviewed [29], and a unified criterion for vaccine efficacy is warranted for future protocols and should be followed. Moreover, future protocols could also benefit by individuals selection standardization, since the protocols recruited chronically-infected individuals; moreover, individuals with advanced HIV-infection and consequently low CD4+ T cell nadirs may not benefit as much as individuals with less advanced infection, as observed by some authors [5,15]. Thus, clinical trials with individuals with early HIV-1 infection may be relevant since in this condition, HIV-1 does not undergo extensive immunologic pressure (and consequently immunologic escape mutations have not been selected), reservoir size is still small and immune function is not yet compromised [30,31]. DC-based therapeutic vaccines may work better in this situation and possibly provide invaluable data about early HIV-1 infection immunology.

Regarding antigen-choice, possibly future trials should use autologous whole virus. This is strategy is time consuming, but it could stimulate a more complete and efficient response involving all possible viral epitopes. Recombinant viral proteins may be more straightforward to use, but they are costly [32], and this must be taken in consideration if the objective is mass production of DC-based immune therapy.

Laboratory methodologies could also be updated. For example, although ELISPOT is commonly used to evaluate cytotoxicity in vaccine trials [33], it simply measures cytokine production, and usually only IFN-γ, being an indirect marker of CTL activity at best. Future DC trials may benefit from the use of multiparameter flow cytometry to obtain a more accurate panel of CTL- specific response [34].

Finally, perhaps the vaccine alone may not be sufficient to cure HIV-1 infection. As there is intense research going on viral reservoirs, it may need to be combined with other strategies for latent HIV-1 activation and induce and potent and efficient CTL activity to kill HIV-1–infected cells [6,16], since current strategies were not able to reduce viral reservoir levels, at least during the available follow-up observations [35]. To this end, the pharmacological reactivation of HIV-1 has been gaining visibility in the last years as a promising strategy, defined as “kick-and-kill” or “shock-and-kill” to eradicate the virus. The depletion of HIV-1 reservoirs, the main objective of this strategy, employs pharmacological modulation of signaling pathways involved in HIV-1 replication. A recent study [36] performed in Denmark described the use of a combined vaccine-pharmacological strategy to deplete HIV reservoirs: in this study, patients were immunized with six doses of Vacc-4x over 12 weeks, followed by romidepsin administration. In this context, the DC-based immune therapy could play a role, as the authors observed an increase in the activation and enhancement of antigen-specific T-cell immune responses, prior to romidepsin treatment. Other initiatives, such as the Research In Viral Eradication of HIV Reservoirs (RIVER) Protocol are actively inquiring the feasibility of a therapeutic vaccine (although not DC-based) and kick-and-kill combination [37], and their results may provide valuable insights for DC-based protocols.

## 4. Material and Methods

### 4.1. Literature Search Strategy and Study Selection

We searched PubMed and MeSH (Medical Subject Headings) databases. Keywords included “Dendritic Cells” [Mesh] AND “Vaccines” [Mesh] AND “HIV” [Mesh]; therapeutic vaccine hiv dendritic cell; AIDS Vaccines dendritic cells; dendritic cells Follow-Up Studies hiv; dendritic cells Follow-Up Studies hiv vaccine. We also searched clinicaltrials.gov database using the following keywords: hiv dendritic and vaccine dendritic|Exclude Unknown|HIV infections.

We reviewed the retrieved texts to assess if they were reports of clinical trials of therapeutic DC-based vaccines against HIV infection which recruited HIV-infected individuals, naïve or treatment-experienced, of any age and any follow-up length. We included both uncontrolled (no control group) and controlled trials (vaccine against placebo, vaccine versus different immunizations strategies or vaccine against no vaccine). Thus, the trials should have included analytical treatment interruptions (ATI) if they enrolled ART-experienced individuals.

If the study was eligible according to these criteria, we recorded the primary outcome, defined as partial or at least transient response to the vaccine (plasmatic HIV-1 viral load decrease). We also recorded adverse events of the experimental vaccinations, safety and tolerability as secondary outcomes.

Additionally, we extracted details from the protocols followed by the authors—number of enrolled individuals and distribution across experimental groups; number of any individuals removed from analysis; cell isolation and DC maturation protocols; time of DC culture; choice of HIV-1 antigen for vaccine preparation; DC count per vaccine dose; types of culture media and cytokine supplementation; forms and routes of experimental vaccine administration; study follow-up length and chosen methodologies for assessment of immunological response to the vaccine.

Two researchers independently reviewed each study and any disagreements were resolved by discussion between them, until a consensus was reached. The collected data were registered in a standard form containing questions regarding the variables defined above.

### 4.2. Statistical Analysis

We performed a meta-analysis to summarize treatment success rate (proportion of individuals in the treatment group with the event of interest—viral load decrease during ATI) among individuals vaccinated during experimental protocols. The treatment success rate was chosen as effect measure because most protocols were uncontrolled (i.e., no placebo arm was enrolled).

Briefly, treatment success rates (described as proportions), were logit-transformed for obtaining the polled treatment success rate, which was back-transformed to a proportion representing the overall quantity of (partial) positive response to the experimental vaccines when considering the total number of vaccinated individuals. Heterogeneity among studies was assessed by I^2^ measure. We chose a random effects meta-analysis model [38] because we considered reasonable that the treatment success rate (the “effect size” in our case) varied across studies, since they were performed in different populations, with different DC collection and maturation procedures, and so on. Thus, following initial meta-analysis, we conducted a meta-regression to assess if these protocols characteristics would explain differences in treatment success rate among them.

To this end, we first conducted univariate regression models to pre-select variables for inclusion on a final, best-fit multivariate meta-regression model with mixed effects. Any variable whose estimated linear meta-regression coefficient had an uncorrected *p*-value <0.30 was pre-selected (the *p*-value for each coefficient tests the null hypothesis that it’s coefficient is equal to zero—no influence over the overall treatment success rate). The level of significance (α) was set at 0.05 for the variables included in the final model. The model fit was assessed by a χ^2^ goodness-of-fit test using the test deviance as an approximation to the χ^2^ distribution with *n*-1 degrees of freedom, in which n is the number of studies included in the analysis. All analyses were performed through R software version 3.2.3 (R core team, Vienna, Austria), using “meta” and “metafor” packages.

## 5. Conclusions

After the systematic review of the literature regarding experimental DC-based anti-HIV-1 immunotherapeutic vaccines, we performed a meta-analysis with 12 selected reports. The vaccines did not work as the clinicians expected, since they had low success rates, but they served as a proof of concept that host immune response could be boosted against HIV-1. There are improvement opportunities for the protocols, which may involve standardization of key steps in trial design: (1) patient selection criteria (approaching their genomics, HLA-typing principally, since it likely plays a role in vaccine response); (2) antigen choice; (3) dendritic cell culture procedures and dosing; (4) unification of immune response “boosting” and success criteria.

## Figures and Tables

**Table 1:** Summary of the 12 studies characteristics that were included in the meta-analysis.

Ref.	Country, City, Year	Inclusion Criteria	Recruited Individuals’ ART Status	Baseline CD4+ T cell count *	Vaccine Doses	Periodicity	Vaccine Administration Form	Anatomical Site (Vaccine Application)	Adverse Effects
[7]	USA, Stanford, 1998	Asymptomatic HIV-1 infection CD4+ T cell counts >350/mm^3^	naive	NR	6	Monthly	intravenous injection	NR	None reported
[8]	Brazil, Recife, 2004	Age of ≥18 years; No current pregnancy; HIV-1 asymptomatic seropositivity for ≥1 year; ART-naive for at least 6 months prior to enrollment; Hemoglobin ≥10 g/dL and platelets ≥100,000	naive	554 ± 174	3	Biweekly	subcutaneous injection	left and right axillary and inguinal areas	Increase in the size of peripheral lymph nodes
[9]	Spain, Barcelona, 2005	Asymptomatic HIV-1 infection; Baseline and nadir CD4+ T cell counts >500 cells/mL; Baseline pre-ART PVL >5000 copies/mL; PVL <20 copies/mL for at least 104 weeks while on ART	experienced	754 ± 36	5	Every six weeks	subcutaneous injection	NR	Flu-like reactions
[5]	Japan, Tokyo, 2006	Undetectable viral loads (PVL <50 copies/mL) for 1 year on ART	experienced	396 (337–504)	6	Biweekly	subcutaneous injection	axillary areas	Subcutaneous bleeding or erythema at injection site General malaise
[10]	USA, Boston and New York, 2009	PVL ≤400 copies/mL and CD4+ T cell counts ≥400/mm^3^ for at least 3 months prior recruitment; PVL <50 copies/mL at screening	experienced	664 (NR)	3	Weeks 3, 7 and 15	subcutaneous injection	inner aspect of the arm, 6–12 cm from the axilla	Episodes of thrombocytopenia in a patient and neutropenia in another
[13]	Denmark, Copenhagen and Hvidovre, 2009	Asymptomatic HIV-1 infection; CD4+ T cell counts ≥300/mm^3^; Absence of other chronic diseases; 1000 < PVL < 100,000 copies/mL; Presence of HLA-A * 0201 allele	experienced	565 (355–982)	4	Biweekly, last dose after four weeks	subcutaneous injection	left and right axillary areas	None reported
[11]	Spain, Barcelona, 2011	Asymptomatic HIV-1 infection; ART-naive for at least two years before enrollment; Baseline CD4+ T cell counts >450 cells/mm^3^; Nadir CD4+ T cell counts >350 cells/mm^3^; PVL >10,000 HIV-1 copies/mL	naive	647 (532–776)	3	Biweekly	subcutaneous injection	NR	Asymptomatic enlargement of local lymph nodes Flu-like symptoms
[15]	Belgium, Brussel and Netherlands, Rotterdam, 2012	Patients on ART; PVL ≤50 copies/ml and CD4+ T cell counts ≥500/mm^3^ for a period of at least 3 months prior to enrollment; Nadir CD4+ T-cell count >300/mm^3^	experienced	610 (500–960)	4	Monthly	subcutaneous and intradermal injection	antero-median side of an arm or a thigh	Tonsillitis episode
[12]	Spain, Barcelona, 2013	Asymptomatic chronic HIV-1 infection; Baseline CD4+ T cell count >450 cells/mm^3^; Nadir CD4+ T cell count >350 cells/mm^3^; Undetectable PVL(<50 copies/mL) on ART	experienced	702 (568–900)	3	Biweekly	subcutaneous or intradermal injection	upper-inner part of both arms	Lymph node enlargement, erythema and flu-like symptoms
[14]	USA, Dallas, 2014	Asymptomatic HIV-1 infection; Baseline CD4+ T cell count >500 cells/mm^3^; Baseline PVL <50 copies/mL and within the previous 3 months while on ART; Nadir CD4+ T cells count ≥300 cells/mm^3^	experienced	670 (553–832)	4	Every 4 weeks	subcutaneous injection	upper and lower extremities	None reported
[6]	USA, Pittsburgh, 2015	CD4+ T cell count ≥300 cells/mm^3^; 3000 < PVL < 100,000 copies/mL	naive	486 (377–881)	4 (3 doses while on ART, 1 dose after ATI)	Biweekly	subcutaneous injection	upper medial area of the arm (bilaterally)	Mild-to-moderate inflammation at the injection site; Two individuals experienced severe pruritus and pain at the injection site
[16]	USA, Philadelphia and Canada, Montreal, 2016	PVL ≤200 copies/mL for at least 3 months prior to enrollment; PVL <50 copies/mL at screening; CD4+ T cell count ≥450 cells/mm^3^; Nadir CD4+ T cell count ≥200 cells/mm^3^; Pre-ART (within 3 months) plasma for virus isolation availability	experienced	632 (513–765)	4	Every 4 weeks	intradermal injection	axillary lymph node	Mild local injection site reactions

ART—antiretroviral therapy; ATI—analytical treatment interruption; Baseline—period before ART start; PVL—plasmatic HIV-1 viral load; NR—not reported; * values expressed as mean ± standard deviation or median (interquartile range).

**Table 2:** Vaccine response criteria of the 12 studies and sample size breakdown for the estimation of global treatment success rate from dendritic cell-based experimental anti-HIV vaccines.

Ref.	Country, City, Year	Vaccine Response Criterion	Enrolled	Placebo Arm	Comparator Arm	Vaccine Arm	Removed from Analysis *n*	Responders *n*	Non-Responders *n*	Study Follow-Up Length
[7]	USA, Stanford, 1998	Any PVL decrease	6	0	0	6	0	0	6	40 weeks
[8]	Brazil, Recife, 2004	>90% PVL decrease by 1 year	20	0	0	18	2	8	10	1 year
[9]	Spain, Barcelona, 2005	PVL decrease of 0.5 log10 copies/mL 24 weeks after vaccination	18	6	0	12	0	4	8	24 weeks
[5]	Japan, Tokyo, 2006	PVL decrease of 0.5 log10 copies/mL	4	0	0	4	0	0	4	12 weeks
[10]	USA, Boston and New York, 2009	Average of the last two scheduled PVL evaluations during weeks 10–13 of ATI ≤5000 copies/mL	29	0	15	14	1	4	9	12 weeks
[13]	Denmark, Copenhagen and Hvidovre, 2009	A PVL decrease of 1.08 log10 copies/mL was the most pronounced change among responders	12	0	0	12	0	5	7	40 weeks
[11]	Spain, Barcelona, 2011	PVL decrease of 0.5 log10 copies/mL 24 weeks after vaccination	24	12	0	12	4	4	4	48 weeks
[15]	Belgium, Brussel and Netherlands, Rotterdam, 2012	Remaining off ART at 96 weeks following ATI	17	0	0	17	0	6	11	110 weeks
[12]	Spain, Barcelona, 2013	Post-vaccination PVL decrease ≥1 log	36	12	0	24	2	12	10	48 weeks
[14]	USA, Dallas, 2014	ATI maximum PVL <5 log10 copies/mL	19	0	0	19	1	9	9	48 weeks
[6]	USA, Pittsburgh, 2015	ATI PVL decrease >0.4 log10 copies/mL	11	0	0	11	1	3	7	48 weeks
[16]	USA, Philadelphia and Canada, Montreal, 2016	PVL in the vaccine arm is reduced by at least 1.1 log10 copies/mL	54	17	0	37	2	0	35	2 years

ART—antiretroviral therapy; ATI—analytical treatment interruption; PVL—plasmatic HIV-1 viral load; NR—not reported.

**Table 3:** Summary of technical differences among the protocols of the 12 studies that were included in the meta-analysis.

Ref.	Loaded Molecules	Loaded Molecules (Summarized)	Dendritic Cell Number	Culture Medium	Days in Culture
[7]	Gag (residues 77 to 85, SLYNTVATL motif), Env (residues 120 to 128, KLTPLCVTL motif and residues 814 to 823, SLLNATDIAV motif) and Pol (residues 956 to 964, LLWKGEGAV motif; residues 464 to 472, ILKEPVHGV motif and residues 267 to 277, VLDVGDAYFSV motif) peptides from recombinant HIV-1 MN gp160 polypeptide	peptides	2–8 × 10^6^ (immature DCs)	RPMI-1640	2
[8]	AT2 (chemically)-inactivated autologous virus	whole virus	6 × 10^7^	CellGro^®^ DC Medium	7
[9]	Heat-inactivated autologous virus	whole virus	2 × 10^6^	MCM	8
[5]	Gag (residues 28 to 36, KYKLKHIVW and KYRLKHIVW motifs; residues 296 to 306, RDYVDRFYKTL motif), Nef (residues 138 to 147, RYPLTFGWCF and RFPLTFGWCF motifs) and Env (residues 584 to 594, RYLRDQQLLGI and RYLQDQQLLGI motifs) peptides	peptides	0.7–1.8 ×10^6^	RPMI-1640	7
[10]	Recombinant virus produced by a canarypox vector (ALVAC vCP1452)	whole virus	1.5–6 × 10^6^	MCM	6
[13]	HLA A*0201-binding peptides (Gag, Pol, Env, Vpu and Vif)	peptides	1 × 10^7^	X-VIVO™ 15	8
[11]	Heat-inactivated autologous virus	whole virus	8 × 10^6^	X-VIVO™ 15	7
[15]	Mature DCs were electroporated with mRNA derived from pGEM-sig-Tat-DC-LAMP, pGEM-sigRev-DC-LAMP, pGEM-sig-Nef-DC-LAMP and pST1-sig-Gag-DCLAMP plasmids for peptides expression	mRNA (by electroporation)	1 × 10^7^	X-VIVO™ 15	7
[12]	Heat-inactivated autologous virus	whole virus	2 × 10^6^	X-VIVO™ 15	7
[14]	Viral epitopes from Gag (17 to 35, and 253 to 284 residues), Nef (66 to 97 and 116 to 145 residues) and Pol (residues 325 to 355) lipopeptides	peptides	15 × 10^6^	CellGro^®^ DC Medium	3
[6]	Autologous CD4+ T cells which had been superinfected with endogenous inactivated HIV-1 with psoralen and UVB irradiation	peptides (indirectly)	1 × 10^7^	CellGro^®^ DC Medium	6
[16]	Mature DCs were electroporated with autologous HIV-1 Gag, Nef, Rev, and Vpr mRNA for peptides expression	mRNA (by electroporation)	1.2 × 10^7^	Not reported	7

AT2—aldrithiol-2; CellGro^®^—registered trademark from CellGenix (Freiburg im Breisgau, Germany); DCs—dendritic cells; RPMI—Roswell Park Memorial Institute medium; X-VIVO™—trademark from Lonza (Basel, Switzerland).

**Table 4:** Summary of cytokine supplementation of the protocols of the 12 studies that were included in the meta-analysis.

Ref.	DC Maturation Procedure								
	GM-CSF Supplementation	IFN-α Supplementation	IFN-γ Supplementation	IL-1β Supplementation	IL-4 Supplementation	IL-6 Supplementation	PGE2 Supplementation	TNF-α Supplementation	Other Molecules Supplementation
[7]	No	No	No	No	No	No	No	No	-
[8]	Yes	No	No	Yes	Yes	Yes	No	Yes	-
[9]	Yes	Yes	No	Yes	Yes	Yes	Yes	Yes	-
[5]	Yes	No	No	No	Yes	No	No	Yes	-
[10]	Yes	Yes	No	Yes	Yes	Yes	Yes	Yes	-
[13]	Yes	No	No	Yes	Yes	Yes	Yes	Yes	-
[11]	Yes	No	No	Yes	Yes	Yes	No	Yes	-
[15]	Yes	No	No	Yes	Yes	Yes	Yes	Yes	-
[12]	Yes	No	No	Yes	Yes	Yes	Yes	Yes	-
[14]	Yes	Yes	No	No	No	No	No	No	LPS
[6]	Yes	Yes	Yes	Yes	Yes	No	No	Yes	polyinosinic:polycytidylic acid
[16]	No	No	Yes	No	No	No	Yes	Yes	CD40L

GM-CSF—granulocyte-macrophage colony-stimulating factor; IFN-α—interferon alpha; IFN-γ—interferon gamma; IL-1β—interleukin 1 beta; IL-4—interleukin 4; IL-6—interleukin 6; LPS—lipopolysaccharide; MCM—monocyte conditioned medium; PGE2—prostaglandin E2; TNF-α—tumor necrosis alpha.

**Table 5:** Univariate analysis of factors and decision to include into multivariate analysis. The objective was to evaluate association with treatment success rate of dendritic cell-based experimental anti-HIV vaccines.

Variable	Linear Univariate Meta-Regression Coefficient	Coefficient Standard Error	*p*-Value	Decision
Antigen:				
whole virus	Reference	-	-	
mRNA	−0.8419	0.5442	0.1219	Pre-selected
peptides	−0.2937	0.3993	0.4621	
Baseline (pre-vaccine) CD4+ T cell counts:				
more than 700 cells/mm^3^	Reference	-	-	Pre-selected
between 600 and 700 cells/mm^3^	−0.4542	0.4405	0.3025	
between 500 and 600 cells/mm^3^	−0.0367	0.5174	0.9434	
less than 500 cells/mm^3^	−0.9804	0.7178	0.1720	
Days in culture (DCs maturation)	−0.0062	0.1118	0.9558	Not pre-selected
DC maturation culture medium:				
RPMI-1640	Reference	-	-	
CellGro^®^	2.8743	0.8972	0.0014	
MCM	2.2890	0.9458	0.0155	Pre-selected
X-VIVO™ 15	2.8780	0.8836	0.0011	
GM-CSF supplementation (yes or no)	3.1400	1.0378	0.0025	Pre-selected
IFN-α supplementation (yes or no)	−0.1512	0.3660	0.6795	Not pre-selected
IFN-γ supplementation (yes or no)	−1.2007	0.6481	0.0639	Pre-selected; removed due to collinearity
IL-1β supplementation (yes or no)	0.5968	0.5336	0.2633	Pre-selected
IL-4 supplementation (yes or no)	0.3529	0.5235	0.5002	Not pre-selected
IL-6 supplementation (yes or no)	0.5672	0.4479	0.2053	Pre-selected; removed due to collinearity
Number of DCs per vaccine dose	−0.0032	0.0408	0.9369	Not pre-selected
Number of vaccine doses	−0.5006	0.2386	0.0359	Pre-selected; removed due to collinearity
Periodicity of vaccine doses (biweekly or every four weeks or more)	0.4793	0.3577	0.1802	Pre-selected; removed due to collinearity
PGE2 supplementation (yes or no)	−0.1765	0.3592	0.6231	Not pre-selected
TNF-α supplementation (yes or no)	−0.1830	0.4887	0.7081	Not pre-selected

DC—dendritic cell; GM-CSF—granulocyte-macrophage colony-stimulating factor; IFNα—interferon alpha; IFNγ—interferon gamma; IL1β—interleukin 1 beta; IL4—interleukin 4; IL6—interleukin 6; PGE2—prostaglandin E2; TNFα—tumor necrosis alpha.

**Table 6:** Multivariate analysis for assessment of which factors were associated with treatment success rate of dendritic cell-based experimental anti-HIV vaccines.

Variable	Linear Multivariate Meta-Regression Coefficient	95% Confidence Interval	*p*-Value
(Model Intercept)	−3.4826	(−6.5475)–(−0.4176)	0.0259
Antigen:			
whole virus	Reference	-	-
mRNA	−0.6289	(−2.1234)–0.8657	0.4095
peptides	1.0689	(−3.2088)–5.3466	0.6243
Baseline (pre-vaccine) CD4+ T cell counts:			
more than 700 cells/mm^3^	Reference	-	-
between 600 and 700 cells/mm^3^	−0.1513	(−1.318)–1.0155	0.7994
between 500 and 600 cells/mm^3^	−1.5793	(−6.0215)–2.8629	0.4859
less than 500 cells/mm^3^	−3.4955	(−12.266)–5.2751	0.4347
GM-CSF supplementation (yes or no)	3.712	(−3.8567)–11.2806	0.3364
DC maturation culture medium:			
RPMI-1640	Reference	-	-
CellGro^®^	−1.147	(−10.2552)–7.9612	0.805
MCM	−3.4024	(−16.7094)–9.9046	0.6163
X-VIVO™ 15	−2.5523	(−15.9238)–10.8191	0.7083
IL-1β supplementation (yes or no)	2.4969	(−6.0232)–11.01	0.5657

Model deviance = 0.81; goodness-of-fit test with 11 degrees of freedom *p*-value = 0.99 (which indicates good fit).
